# Serological diagnostics of Lyme borreliosis: comparison of assays in twelve clinical laboratories in Northern Europe

**DOI:** 10.1007/s10096-019-03631-x

**Published:** 2019-08-09

**Authors:** Malin Lager, Ram B. Dessau, Peter Wilhelmsson, Dag Nyman, Guro F. Jensen, Andreas Matussek, Per-Eric Lindgren, Anna J. Henningsson, The ScandTick Biobank Study Group, Haitham Baqir, Lena Serrander, Marcus Johansson, Ivar Tjernberg, Ingerid Skarstein, Elling Ulvestad, Nils Grude, Anne-Berit Pedersen, Anders Bredberg, Renate Veflingstad, Linda Wass, Josefin Aleke, Marika Nordberg, Clara Nyberg, Linda Perander, Christina Bojesson, Emma Sjöberg, Åslaug R. Lorentzen, Randi Eikeland, Sølvi Noraas, Gunnel AL Henriksson, Gábor Petrányi

**Affiliations:** 1 Division of Clinical Microbiology, Laboratory Medicine, Jönköping Region Jönköping County, Sweden and Department of Clinical and Experimental Medicine, Linköping University, Ryhov County Hospital, SE-551 85 Jönköping, Sweden; 2 Division of Medical Microbiology, Department of Clinical and Experimental Medicine, Linköping University, Linköping, Sweden; 3 Department of Clinical Microbiology, Slagelse Hospital, Slagelse, Denmark; 4 The Åland Group for Borrelia Research, Åland, Mariehamn, Finland; 5 Department of Medical Microbiology, Sørlandet Hospital, Kristiansand, Norway; 6 Karolinska University Laboratory, Stockholm, Sweden; 7 Division of Clinical Microbiology, Department of Laboratory Medicine, Karolinska Institutet, Karolinska University Hospital Huddinge, Stockholm, Sweden; 8 Division of Clinical Microbiology, Department of Clinical and Experimental Medicine, Linköping University Hospital, Linköping, Sweden

**Keywords:** *Borrelia burgdorferi* sensu lato, Serology, Laboratory diagnosis, Antibodies

## Abstract

Lyme borreliosis (LB), caused by spirochetes belonging to the *Borrelia burgdorferi* sensu lato complex, is the most common tick-borne infection in Europe. Laboratory diagnosis of LB is mainly based on the patients’ medical history, clinical signs and symptoms in combination with detection of *Borrelia*-specific antibodies where indirect enzyme-linked-immunosorbent assay (ELISA) is the most widely used technique. The objective of the study was to evaluate and compare the diagnostic accuracy (sensitivities and specificities) of serological tests that are currently in use for diagnosis of LB in clinical laboratories in No**r**thern Europe, by use of a large serum panel. The panel consisted of 195 serum samples from well-characterized and classified patients under investigation for clinically suspected LB (*n* = 59) including patients with Lyme neuroborreliosis, Lyme arthritis, acrodermatitis chronica atrophicans, erythema migrans or other diseases (*n* = 112). A total of 201 serum samples from healthy blood donors were also included. The panel (396 serum samples altogether) was sent to 12 clinical laboratories (using five different ELISA methods) as blinded for group affiliation and the laboratories were asked to perform serological analysis according to their routine procedure. The results from the study demonstrated high diagnostic concordance between the laboratories using the same diagnostic assay and lower diagnostic concordance between laboratories using different diagnostic assays. For IgG, the results were in general rather homogenous and showed an average sensitivity of 88% (range 85–91%) compared to IgM which showed lower average sensitivity of 59% (range 50–67%) and more heterogeneous results between assays and laboratories.

## Introduction

Lyme borreliosis (LB) is the most common tick-transmitted disease in Europe and is caused by spirochetes belonging to the *Borrelia burgdorferi* sensu lato (s.l.) complex [1]. The annual incidence varies from 1/100,000 to > 100/100,000 inhabitants in different countries in Europe [2, 3]. Clinical manifestations of LB include erythema migrans (EM), Lyme neuroborreliosis (LNB), acrodermatitis chronica atrophicans (ACA) and Lyme arthritis (LA) [4]. Diagnosis of LB, except for EM which is considered as a clinical diagnosis, is based on the presence of typical symptoms and signs, the patients’ medical history in combination with laboratory evidence of borrelia infection. In clinical practice, serological detection of *Borrelia-*specific antibodies by enzyme-linked immunosorbent assay (ELISA) is widely used, sometimes supplemented by immunoblot in order to increase the specificity and the positive predictive value [5]. However, this two-tiered testing approach is expensive, time-consuming and laborious and may not be necessary with modern ELISAs that are based on synthetic or recombinant antigens [6]. Modern ELISA methods have high analytical sensitivity and specificity, besides being inexpensive and easy to perform [5]. However, there are some limitations in clinical interpretation due to biological aspects that need to be taken into consideration. For instance, the natural delay in antibody response in relation to onset of symptoms in LB may influence the diagnostic sensitivity in early LB [7], and possible IgM cross-reactivity between antigens of pathogens within the same genus, but also in different genera, may lead to false-positive results [8, 9]. The long-term persistence of antibodies after a *Borrelia* infection and the high seroprevalence in the healthy populations in endemic areas may also have impact on the clinical diagnostic specificity, since it can be complicated to distinguish an active from a previous *Borrelia* infection [10–12]. An investigation from 2011, based on a survey alone, summarized the different methods used at 43 laboratories in Sweden, Norway, Denmark and Finland [13]. The survey showed differences regarding methods/combinations of methods, strategies (one-step or two-step), choice of assays and cut-off values between laboratories and countries. This study, together with many other studies evaluating and surveying the diagnostic assays for serological testing, is a good example showing the lack of uniformed methods used for detection of LB and the need of further development of recommendations for interpretation and reporting in order to achieve more consistent laboratory diagnostics of LB in Europe. Data to support the two-step strategy in a European clinical setting is ambiguous [6, 13, 14]. The objective of this present study was to evaluate and compare the diagnostic accuracy (sensitivities and specificities) of several serological ELISA methods that are currently in use for LB diagnosis in clinical laboratories in Northern Europe (including Sweden, Norway, Denmark and the Åland Islands, Finland), by using a large and well-characterized panel of sera from patients and controls.

## Material and methods

### Study design

A cross-sectional study design was used to create a panel of serum samples representative for patients referred to specialist clinics for suspected LB. The study panel contained 396 serum samples, including 195 serum samples from clinically and laboratory well-characterized patients (> 18 years of age) under investigation for clinically suspected LB in Jönköping County, in the municipality of Kristiansand and on the Åland Islands and 201 blood donors. All patient samples were prospectively included in the study and then retrospectively classified based on the patients’ medical records. The 195 serum samples were consecutively collected from patients referred to the Department of Infectious Diseases, County Hospital Ryhov, Region Jönköping County, Sweden (2013–2017), Department of Neurology, Sørlandet Hospital, Kristiansand, Norway (2015–2017) or the Department of Medicine, Åland Central Hospital and Bimelix Laboratory, Mariehamn, Åland, Finland (2014–2017) for suspected LB manifestations (LNB, ACA, LA, EM). Medical records were reviewed independantly by two experienced phycisians specialised in either infectious diseases, clinical microbiology or neurology, and the patients were then classified as described below. The samples from blood donors were collected at the Department of Transfusion Medicine, Laboratory Medicine, Region Jönköping County, Sweden (2016–2017).

### Participants

Serum and cerebrospinal fluid (CSF) were collected when the patients were referred to the specialist clinics for investigation of suspected LB manifestations and the samples were analysed according to the local standard procedure, used at the respective laboratory at the hospitals recruiting the patients, including both CSF cell count, detection of *Borrelia*-specific antibodies and calculation of intrathecal antibody index (AI). The serological assays used at the three recruiting hospitals were IDEIA Lyme Neuroborreliosis test (Oxoid, Hampshire, UK) and Enzygnost Borrelia Lyme IgM/IgG (Siemens/DADE Behring, Marburg, Germany) in Sweden, Enzygnost Borrelia Lyme IgM/IgG (Siemens/DADE Behring) in Norway and Immunogenics® C6 LYME ELISA ™kit, IgM/IgG (Immunetics, Inc., Boston, MA) and RecomWell Borrelia IgM/IgG (Mikrogen, Neuried, Germany) on the Åland Islands. Manifestations like LA and ACA were confirmed by *Borrelia*-specific PCR in addition to the serological testing, while the diagnosis of EM was solely based on the physician’s clinical assessment [2]. All serum samples were taken before treatment and only one sample per patient was included. The blood donors had stated that they were healthy and a health declaration was completed before the blood donation.

**Fig. 1:**
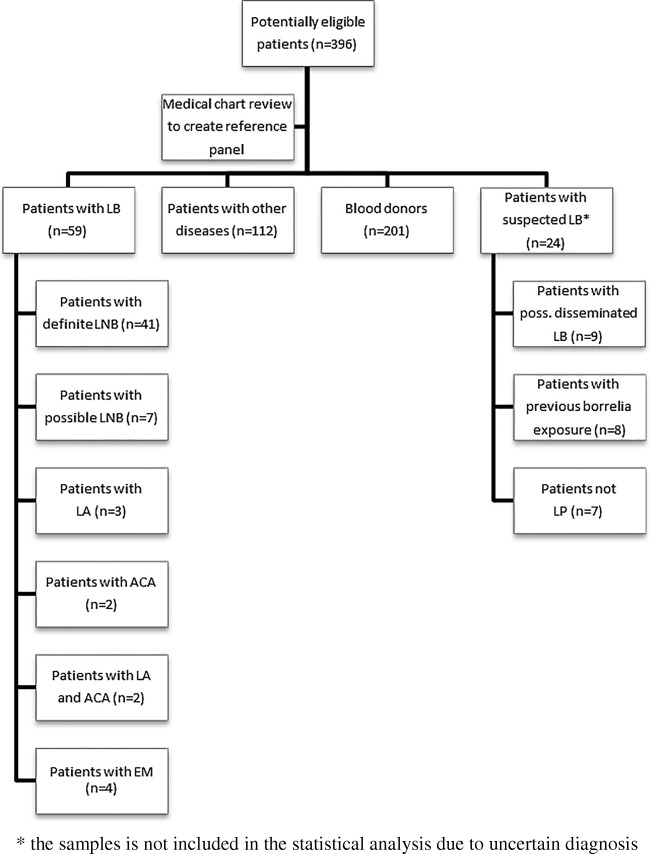
A flow chart demonstrating the inclusion and classification process in the study. LA = Lyme arthritis, ACA = acrodermatitis chronica atrophicans, EM = erythema migrans, LB = Lyme borreliosis, LNB = Lyme neuroborreliosis, LP = lumbar puncture. “*” The samples is not included in the statistical analysis due to uncertain diagnosis

Based on both laboratory results and by review of medical charts, the patients were retrospectively classified into four groups, (1) LB patients with manifestations including definite LNB, LA, EM or ACA (*n* = 59), (2) patients with other diseases (*n* = 112), (3) blood donors (*n* = 201) and (4) suspected LB (*n* = 24) (Fig. 1). The latter group presented with symptoms and signs that did not fulfill the criteria for any of the LB groups and they were referred for evaluation at the specialist clinics because of their seropositivity. However, they were not included in the statistical analysis since the patients in this group were difficult to evaluate and classification was uncertain. A flow chart demonstrating the inclusion and classification process is shown in Fig. 1. The criteria for classification are shown in Table 1 and age at time for inclusion and sex for all four groups together with the major clinical symptoms and signs from patients with other diseases, not classified as LB patients, are shown in Table 2.

**Table 1:** Clinical classification criteria for the four study groups

Classification	Criteria
1. LB patients (*n* = 59)	Definite LNB (*n* = 41)^b^
	1. Neurological symptoms indicative of LNB without other plausible reasons
	2. Pleocytosis in CSF^a^
	3. Intrathecal production of anti-*Borrelia* antibodies (IgM and/or IgG)
	Possible LNB (*n* = 7)^b^
	Criteria 1 and 2 above fulfilled
	LA (*n* = 3)
	Clinical signs of arthritis, pleocytosis and detection of *Borrelia*-specific DNA in synovial fluid
	ACA (*n* = 2)
	Clinical signs compatible with ACA and detection of *Borrelia*-specific DNA in skin biopsy
	LA + ACA (*n* = 2)
	Criteria for both LA + ACA fulfilled
	Erythema migrans (*n* = 4)^c^
	Recent tick-bite and typical skin rash >5 cm in diameter (assessed by a physician)
2. Other diseases (*n* = 112)	Patients not meeting the criteria for definite LNB, possible LNB, LA, ACA or EM but with either specific diagnosis of previous LNB, other CNS illness (such as TBE (*n* = 1) and enterovirus meningitis (*n* = 1)) or no CNS illness
3. Blood donors (*n* = 201)	Blood donors who completed a health declaration and stated no current symptoms or signs of disease
4. Suspected LB patients (*n* = 24)^d^	Possible disseminated LB^e^ (*n* = 9)
	Patients with symptoms not explained by any other disease and with significantly elevated and rising IgG antibody titer in serum. In some cases also intrathecal antibody production in CSF, but no pleocytosis nor increased levels of CXCL13 (<20 pg / mL) in CSF. Probable (visited an endemic area) or observed tick-bite. Good response to antibiotic treatment (amoxicillin or doxycycline).
	Previous infection (*n* = 8)
	Patients with intrathecal antibody production in CSF but without signs of pleocytosis or increased levels of CXCL13 in CSF (>20 pg / mL). The patients received no antibiotic treatment
	Not LP (*n* = 7)
	Patients not classified due to lack of lumbar puncture and CSF analysis

^a^Total cell count ≥ 5 × 10^6^/L in CSF

^b^Classified in accordance with European guidelines [2, 15]

^c^Classified in accordance with European guidelines [2]

^d^All but one of the samples, recruited in Jönköping, was from patients located on the Åland islands

^e^Patients not classified as LNB, ACA, LA, EM, lymphocytoma or carditis

*LB* = Lyme Borreliosis, *LNB* = Lyme neuroborreliosis, *CSF* = cerebrospinal fluid, *LA* = Lyme arthritis, *ACA* = Acrodermatitis chronica atrophicans, *EM* = Erythema migrans, *CNS* = central nervous system, *TBE* = tick-borne encephalitis, *Dissem. LB* = disseminated Lyme borreliosis, *LP* = lumbar punctured

**Table 2:** Age at time of inclusion and sex for the 396 patients together with the major clinical symptoms and signs from patients with other diseases, not classified as LB patients

On admission	
Patients with LB (*n* = 59)	
Age, median years (range)	55 (21–85)
Symptom duration, range in days	1–120
*Gender*	
Female, *n* (%)	33 (56)
Male, *n* (%)	26 (44)
Patients with other diseases (*n* = 112)	
Age, median years (range)	56 (18–89)
Symptom duration, range in days	3–3650
*Gender*	
Female, *n* (%)	59 (53)
Male, *n* (%)	53 (47)
*Major clinical features*	
Headache, *n* (%)	43 (38.4)
Fatigue, *n* (%)	33 (29.5)
Myalgia/joint pain, *n* (%)	35 (31.6)
Pain/radiating pain, *n* (%)	22 (19.6)
Sensory disorders, *n* (%)^a^	29 (25.9)
Neck pain, *n* (%)	23 (20.5)
Facial nerve palsy, *n* (%)	9 (8.0)
Back pain, *n* (%)	12 (10.7)
Vertigo, *n* (%)	14 (12.5)
Memory disorders/concentration difficulty, *n* (%)	15 (13.4)
Skin rash (not assessed as EM)	3 (2.7)
Blood donors	
Age, median years (range)	47 (20–68)
*Gender*	
Female, *n* (%)	68 (33)
Male, *n* (%)	133 (66)
Patients with suspeceted LB	
Age, median years (range)	58 (25–78)
Symptom duration, range in days	28–730
*Gender*	
Female, *n* (%)	11 (46)
Male, *n* (%)	13 (54)

^a^Including symptoms like hyperacusia, photofobia, dysacusia, diplopia, vision loss, aphasia, numbness and itching

### Test methods

The study involved 12 clinical laboratories (referred to as laboratory 1–12) located in Sweden (*n* = 6), Norway (*n* = 4), Denmark (*n* = 1) and the Åland Islands, Finland (*n* = 1) using commercial borrelia serology assays quite representative for clinical laboratories in these countries. The study panel, consisting of frozen serum samples, was sent on dry ice and blinded for group affiliation to the laboratories, but also blinded to the coordinating laboratory, which was the Laboratory of Clinical Microbiology, Laboratory Medicine, Region Jönköping County, Sweden (CMLJ). The participating laboratories were asked to analyse the samples according to their routine procedure and the results together with the method descriptions were reported to the CMLJ for compilation. The serum samples in the panel were analysed according to the laboratories’ diagnostic routine procedure. All laboratories based their primary diagnostics on ELISA, and assays from five different manufacturers were used. Participating laboratories, diagnostic assays, abbreviations for the different diagnostic assays used in this manuscript, manufacturers, reference intervals and cut-offs together with units are presented in Table 3. In the qualitative comparison, the cut-off value from each laboratory was used to establish results as positive or negative. In the quantitative comparison, the cut-off values were not taken into consideration. The serological results were reported as positive, borderline or negative. Borderline results were regarded as positive in the statistical analyses.

**Table 3:** A summary of the different diagnostic methods used for detection of *B. burgdorferi* s.l. at 12 laboratories (1–12) in Sweden, Norway, Denmark and Finland

**Diagnostic assays for IgM** ^**1**^			**Cut-off values**			
**Diagnostic assay/manufactures/antigens** ^**2**^	**Abbreviation**	**Laboratory**	**Negative**	**Borderline**	**Positive**	**Units**
Liaison Borrelia IgM Quant (DiaSorin, Saluggia, VC, Italy)	Liaison IgM Quant	1	< 16.0	≥ 16.0–< 24.0	≥ 24.0	AU/mL
Recombinant OspC from *Ba* PKo and VlsE		2	< 22.0	–	≥ 22.0	
		3	< 30.0	30.0–35.0	> 35.0	
Liaison Borrelia IgM II (DiaSorin)	Liaison IgM II	4	< 0.9	0.9–1.1	> 1.1	Lyme index
Recombinant OspC from *Ba* PKo and VlsE		5	≤ 0.89	0.90–1.09	≤ 1.1	
Enzygnost Borrelia Lyme IgM (Siemens / DADE Behring, Marburg, Germany)	Enzygnost IgM	6	< 2.0	–	≥ 2.0	U/mL
Based on a detergent extract from *Ba* strain PKo		7			Varies between runs but is generally set to 0.3	OD
		8			Mean value of negative control +0.280	U/mL
Anti-Borrelia ELISA, IgM (EuroImmun, Luebeck, Germany)	EuroImmun IgM	9	< 40.0	–	≥ 40.0	RU/mL
Mix of whole-cell antigen extracts from *Bb, Ba* and *Bg*						
RecomWell Borrelia IgM (Mikrogen, Neuried, Germany)	RecomWell IgM	10	< 20.0	20.0–24.0	> 24.0	U/mL
Recombinant OspC, p41/internal, VlsE from *Ba*, *Bg* and *Bb*						
**Diagnostic assays for IgG**			**Cut-off values**			
**Analyse method/manufactures/antigens** ^**2**^	**Abbreviation**	**Laboratory**	**Negative**	**Borderline**	**Positive**	**Units**
Liaison Borrelia IgG (DiaSorin)	Liaison IgG	1	≤ 9.0	≥ 9.0–< 17.0	≥ 17.0	AU/mL
Recombinant Borrelia specific VlsE antigens from *Bg* strain PBi		2	< 10.0	10.0–14.9	≥ 15.0	
		3	< 10.0	10 0–15.0	> 15.0	
		4	< 10.0	10.0–< 15.0	≥ 15.0	
		5	≤ 10.0	10.1–14.9	≥ 15.0	Lyme index
Enzygnost Borrelia Lyme link VlseE/IgG (Siemens / DADE Behring)	Enzygnost IgG	6	< 10.0	–	≥ 10.0	U/mL
Mix of native Borrelia antigens from *Ba* strain PKo and		7			100%	% of cut-off
recombinant VlsE obtained from genospecies *Bb* s.s.,* Bg* and *Ba*		8			Mean value of negative control + 0.150 (5 U/mL)	U/mL
Anti-Borrelia plus VlsE ELISA, IgG (EuroImmun)	EuroImmun IgG	9	< 40.0	–	≥ 40.0	RU/mL
Mix of whole-cell antigen extracts from *Bb, Ba* and *Bg* plus						
VlsE *Bb* (purified recombinant protein)						
RecomWell Borrelia IgG (Mikrogen)	RecomWell IgG	10	< 20.0	20.0–24.0	> 24.0	U/mL
Recombinant p100, OspC, VlsE, p18 from* Ba*, *Bg* and *Bb*						
Immunogenics® C6 LYME ELISA ™kit, IgM/IgG (Immunetics, Inc., Boston, MA)	C6 ELISA	11	< 0.90	0.91–1.09	≥ 1.10	Lyme Index
Synthetic C6 peptide (25 aa) derived from IR_6_ of VlsE *Bb* strain B31		12	< 0.90	0.91–1.09	≥ 1.10	

^1.^The information regarding the Immunogenics® C6 LYME ELISA ™kit, IgM/IgG is presented under diagnostic assays for IgG.

^2.^Ba = *Borrelia afzelii*, Bg = *Borrelia garinii*, Bb s.s. = *Borrelia burgdorferi* sensu stricto

### Data analysis

The R statistic software [16] was used for statistical analysis and graphics. The receiver operating characteristic curve (ROC) analyses were performed using R statistic software, package pROC and mada. The R-package mada is a tool implementing the so-called “Reitsma” method for the meta-analysis of bivariate diagnostic accuracy [17]. For bivariate comparison of sensitivities and specificities, the sROC approach was used. Results outside the 95% confidence regions for fits were considered statistically significant. The statistical comparison of area under curve (AUC) used the command roc.test with the default “Delong” algorithm [18]. The assessed results in the qualitative comparison (positive, borderline or negative) have been established by the participating laboratories, while the quantitative comparison is based on the numerical values reported for each sample from each laboratory.

## Results

### Qualitative comparison

#### Qualitative comparison within the diagnostic assays

**Fig. 2:**
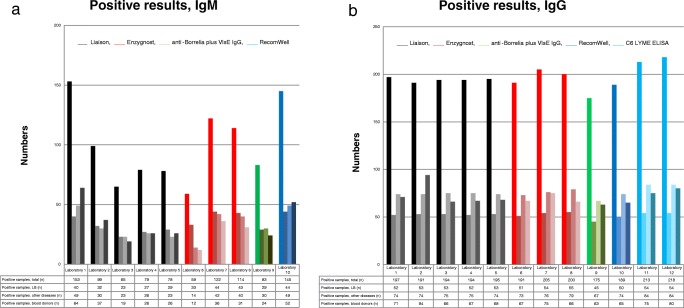
Numbers of positive samples (in each group) per assay for all the 12 laboratories for all 372 samples. The colored bars (black = Liaison assays, red = Enzygnost assays, green = EuroImmun assay, blue = RecomWell assay and light blue = C6 ELISA assays) represent the number of positive results and the different colors represent the different diagnostic assays. Each laboratory is presented according to: bar 1 = positive samples, total, bar 2 = positive samples, LB, bar 3 = positive samples, other diseases and bar 4 = positive samples, blood donors. **a** Illustrate laboratories 1–10 (laboratories 11–12 are shown in plot b) for the IgM assays. **b** Illustrate laboratories 1–12 for the IgG assays, including C6 ELISA. Laboratories 1–3 use the Liaison Borrelia IgM Quant assay

The twelve laboratories used five different assays, where three of them were used at more than one laboratory and will be compared in this section (Table 3). The results in this section are presented as range of positive results (rpr), representing the range between the highest and the lowest number of positive results within a diagnostic assay. The IgM assay showed a heterogenic picture with low correlation both within assays and between assays while the IgG assays showed a more homogeneous picture with high correlation. The rpr in the IgG asays are as follows: (1) Liaison IgG assay (rpr = 191–197), (2) Enzygnost IgG assay (rpr = 191–205) and (3) C6 ELISA (rpr = 213–218) (Fig. 2b). The rpr in the IgM assays are (1) Liaison IgM Quant assay (rpr = 65–153)/Liaison IgM II assay (rpr = 78–79) and (2) Enzygnost IgM assay (rpr = 59–122) (Fig. 2a). The highest number of positive results, for the Liaison IgM assays, was seen in laboratory 1 (rpr = 153) (Fig. 2a) which also reported a rather high number of borderline results. This laboratory also had the highest sensitivity. Laboratory 2 showed the lowest number of borderline results for IgM. The lowest number of positive results for the Enzygnost IgM assay (*n* = 59) (Fig. 2b) was reported from laboratory 6 (in both blood donors and patients with other diseases). This laboratory had adjusted their cut-off value from < 1.0 U/mL, given by the manufacturer, to < 2.0 U/mL in order to decrease the number of false-positive results. This resulted in higher specificity but also lower sensitivity compared to laboratories 7 and 8. Finally, the C6 ELISA showed high correlation between laboratories 11 and 12, and few (7 respective 6) borderline results were reported.

#### Qualitative comparison between the diagnostic assays for IgG and IgM

**Fig. 3:**
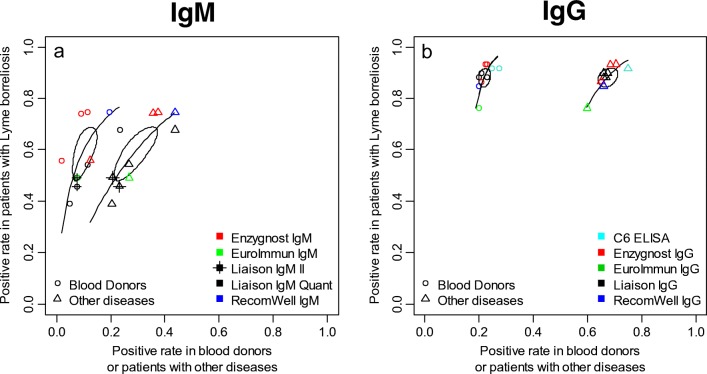
Positive rate for each diagnostic assay (plot **a:** IgM, plot **b:** IgG) in patients with LB, blood donors and patients with other diseases. The elliptic 95% confidence regions on the ROC space are representing the statistical average of the 12 laboratories. The bent curve is the calculated summary ROC. Black = Liaison assays, red = Enzygnost assays, green = EuroImmun assay, blue = RecomWell assay and light blue = C6 ELISA assays

Using bivariate analysis of the sensitivity and specificity, the rate of positive results among patients with LB in all five IgM assays used at the 12 laboratories was low, with an average sensitivity of 59% (range 50–67%) (Fig. 3a, *y*-axis), with a large heterogeneity from 40 to 80% (Fig. 3a, *y*-axis). The positive rate among blood donors for IgM was in average 9.5% (range 7–14%) and among patients with other diseases 28.6% (range 23–35%) in average corresponding to the *x*-axis in Fig. 3a.

Four out of five IgG assays (Fig. 3b) showed high average sensitivity of 88% (range 85–91%) among patients with LB (Fig. 3b, *y*-axis). The positive rate among blood donors for IgG was in average 22% (range 20–24%) and among patients with other diseases 68% (range 35–71%) in average corresponding to the *x*-axis in Fig. 3b. The laboratory using EuroImmun IgG had a lower positive rate compared to the other laboratories, 76% (range 64–85%) among patients with LB (Fig. 3b, *y*-axis), and a slightly higher positive rate for blood donors and patients with other diseases (Fig. 3b, *x*-axis), 20% (range 14–26%) and 60% (range 50–68%), respectively (Fig. 3b, *x*-axis). The choice of control group has large influence on the apparent clinical diagnostic specificity, with a high average positive rate 60% (range 50–68%) in patients with other diseases (Fig. 3b, *x*-axis). The C6 ELISA showed a slightly higher positive rate both among patients with other diseases, 75% (range 66–82%) in average (Fig. 3b, *x*-axis) and blood donors, 26% (range 21–33%) in average (Fig. 3b, *x*-axis). However, the positive rate among patients with LB was comparable to the rest of the diagnostic assays (average sensitivity of 88% (range 85–91%) Fig. 3b, *y*-axis)). The seroprevalences among blood donors in the study ranged from 20 to 27% (Fig. 3b, *x*-axis) between the five diagnostic assays, with a slightly higher number for the C6 ELISA and a slightly lower number for laboratories 7 and 8, using the Enzygnost IgG assay and for laboratory 1 using the Liaison IgG assay (data not shown). Overall, the homogeneity among the different assays is higher for IgG compared to IgM, and by using the test for separate comparison of the sensitivities and specificities, the *p* values for differences regarding IgG are not significant (*p* > 0.20) but highly significant for IgM (*p* < 0.001).

### Quantitative comparison

#### Quantitative comparison within the diagnostic assays

**Fig. 4:**
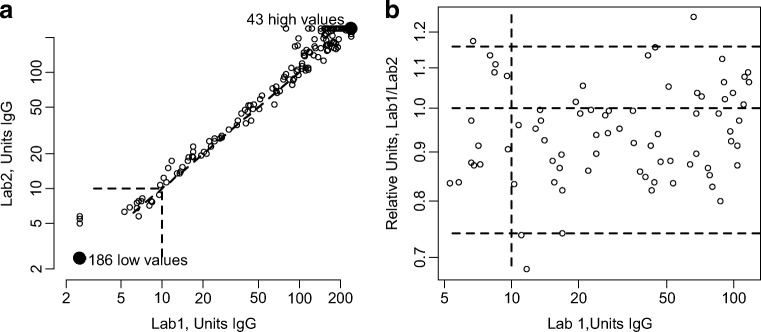
An example of a comparison of the quantitative measurements from two laboratories on 396 samples using the Liaison IgG assay. **a** XY-plot of the two measurements from the same sample for laboratories 1 and 2. **b** The units of the samples from laboratory 1 and the relative units defined as the measurements from laboratory 1 divided by laboratory 2 correspond to a Bland-Altman plot subtracting the logarithm of the measurements. For this plot, only 76 samples, which in both laboratories are in the range between > 5 and ≤ 120 units, are included. Horizontal broken lines represent the 2.5% (lower) and 97.5% (upper) quantiles and the expected equality = 1. The vertical line at 10 units designates the specified cut-off value for the assay

In order to assess the analytical technical performance of the laboratories using the same assay, a quantitative comparison was performed using the numerical values of each sample obtained at each laboratory. Pairwise comparison between the laboratories using the same diagnostic test was established. Figure 4a illustrates a representative example of comparison between laboratories using the same assay, in this case laboratories 1 and 2 for the Liaison IgG assay. The intra-assay correlation displayed good agreement along the diagonal of equal values (Fig. 4a), except for some of the really high values where laboratory 1 tended to have lower results. In this case, no samples around the cut-off were reclassified as positive or negative. All correlation curves for the three assays used at more than one laboratory showed curves comparable to the one shown in Fig. 4a (data not shown). In the Bland-Altman plot (Fig. 4b), the very high and low values are excluded and the horizontal broken line corresponds to the diagonal line in Fig. 4a. The results in Fig. 4b show that the measurement error within the quantitative range of the instrument is lower for laboratory 1, within a 95% range around the equality line of one from 0.74 to 1.16. This inter-assay variation is highly acceptable (and impressive) with a coefficient of variation of 12% and 95% of the values within ± 20% of the mean. The remaining IgG assays and the IgM assays show similar high correlation within the assays (data not shown).

#### Quantitative comparison between the diagnostic assays

**Fig. 5:**
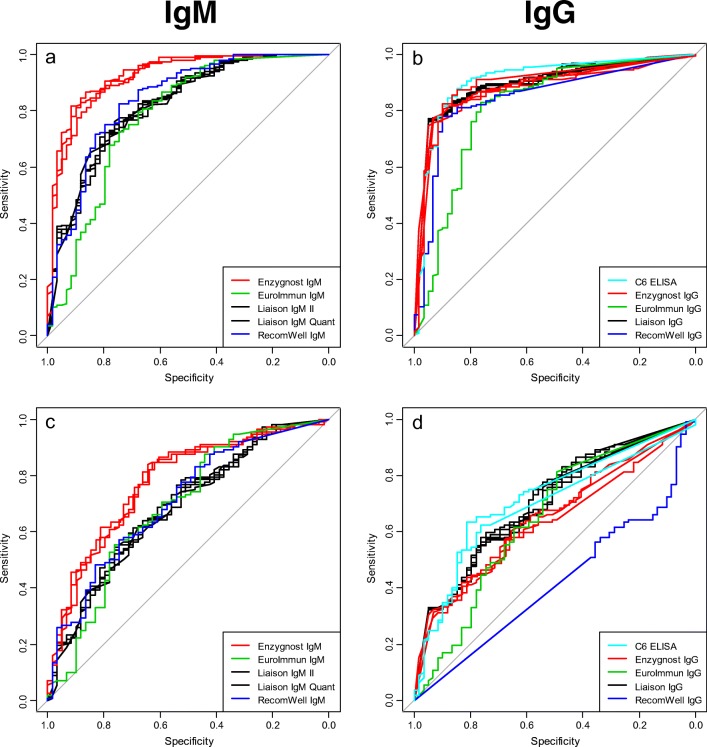
ROC curves of the quantitative results. **a**, **b** Fifty-nine patients with LB compared to 201 blood donors (group 3) for IgM and IgG respectively. **c**, **d** Fifty-nine patients with LB compared to 112 patients with other diseases (group 2) for IgM and IgG, respectively. Black = Liaison assays, red = Enzygnost assays, green = EuroImmun assay, blue = RecomWell assay and light blue = C6 ELISA assays

A ROC curve analysis was performed for all laboratories comparing patients with LB to patients with other diseases and to blood donors. The ROC curve analysis showed that the EuroImmun assay has a relatively low specificity compared to the other assays and that the AUCs are significantly lower when the blood donors are used as negative controls in contrast for both IgM and IgG compared to the Liaison IgG assay (*p* = 0.006) (Fig. 5a, b). For IgM analysis, the Enzygnost IgM assay has the highest AUC compared to the Liaison IgM assay (*p* = 0.001) (Fig. 5a+c). When comparing the patients with LB to the patients with other diseases, the AUCs are lower and more similar, except for the Enzygnost IgM assay which still performs better (*p* = 0.008) (Fig. 5c). The EuroImmun IgG assay has a low AUC of 0.65 (range 0.56–0.74) which is significantly lower than the Liaison IgG assay with a AUC of 0.71 (range 0.63–0.79) (*p* < 0.01) (Fig. 5b). The RecomWell IgM/IgG assay is in line with the Liaison IgM/IgG assay using blood donors as controls (Fig. 5a, b) as well as IgM using patients with other disease as controls (Fig. 5c). However, the RecomWell IgG assay has lower AUC of 0.42 (range 0.33–0.50) compared to the Liaison IgG assay of 0.71 (range 0.63–0.79) (*p* < 0.0001) using patients with other disease as controls (Fig. 5d). The low specificity for the RecomWell IgG assay using patients with other diseases as control may be a result of the high number of positive results at the maximum range of the assay for both patients with LB and other diseases, and therefore the poor performance with an AUC of 0.42 (range 0.33–0.50) (Fig. 5d). These figures also support that the same assays gave the same results in different laboratories.

## Discussion

This study is an attempt to determine if there is any significant variability between and within the diagnostic assays currently in use at clinical laboratories in Northern Europe by using a large and well-characterized panel of serum samples from patients and controls. The results show high intra-assay correlation between the laboratories using the same diagnostic assay (especially for IgG) and lower correlation between laboratories using different diagnostic assays. Both the intra- and inter-assay comparison showed more specific results with high compliance for IgG, and lower for IgM. Interestingly, we found an increased seroprevalence among blood donors compared to previous studies from the same geographical areas [11].

The results within the IgM assays showed more heterogeneity compared to the IgG assays, not only between the different diagnostic assays, but also between laboratories using the same diagnostic assays. This may suggest reproducibility problems in the IgM assays, but is more likely a result of the different cut-off values used in the different diagnostic assays or the fact that some laboratories [1, 7–10] have numerous samples (> 20 samples) with borderline results, which in this study were classified as positive in the statistical analyses. The low specificity for the IgM assays was expected since it is well known that IgM antibodies are less mature and specific than IgG antibodies, and false-positive IgM reactions due to cross-reactivity are difficult to overcome. In everyday practice of clinical microbiology, IgM interpretation may indeed be challenging and should be performed cautiously.

The IgG assays showed high concordance and more homogeneous results both between and within assays. The EuroImmun IgG assay showed a slightly lower sensitivity with no major gain in specificity compared to the other assays. This is in line with previous studies showing a higher sensitivity and specificity for assays based on recombinant antigens compared to whole cell lysate [19]. However, a previous study showed the opposite results with superior sensitivity and negative predictive value in negative tests in combination with low specificity and positive predictive value [20]. Both this study and the one by *Kodym* et al. 2018 include a low number of laboratories, which makes it hard to draw any firm conclusions. However, Kodym et al. [21] suggest that the EuroImmun IgM/IgG assay may serve as a screening test to be used together with a confirming immunoblot. Overall, the serological methods showed high concordance and comparable sensitivity and specificity regarding IgG both within and between assays, while the IgM assays showed more heterogenic and less sensitive results. This implicates that if laboratories were to analyse only *Borrelia*-specific IgG in serum, patients and clinicians were to receive more or less the same test result irrespective of which laboratory that performed the analysis. However, IgM results differ considerably more between laboratories and methods, and our data suggest that IgM testing in serum does not really add any diagnostic value to IgG testing in suspected LB cases, since the sensitivity for IgM is lower (with the possible exception of the Enzygnost Borrelia IgM assay) and results in loss of specificity. Also, the positive rate of IgM in sera from patients with other diseases is higher than among blood donors, illustrating the well-known risk of false-positive reactivities [14]. Taken together, IgM testing in serum samples is a diagnostic tool that is difficult to handle correctly and its value in clinical diagnostics of LB may be questioned. It is important to keep in mind, though, that in this study, we have not included CSF samples or samples from children, and the clinical value of *Borrelia*-specific intrathecal IgM index or IgM testing in pediatric sera cannot be assessed here.

Commercial assays are marketed using different antigens or combinations of antigens. Comparison of diagnostic assays with different antigens will show less analytical correlation. This is consistent with biology since the reactivity to different antigens or antigen combinations will statistically have conditional independence. Antibodies develop differently in different individuals and different assays detect different antibodies, which may result in both strong and weak correlation when no antibody development is measured in one assay and a high reactivity is measured in another assay. It has been shown in reports concerning external quality assurance, round robins where a smaller number of samples are tested in many laboratories to result in some variation between the laboratories [20]. The Enzygnost IgG assay uses a mix of whole cell detergent extract and recombinant VlsE from the three main *B. burgdorferi* s.l. species pathogenic to humans, whereas the Liaison IgG assay, according to kit insert, is based solely on recombinant VlsE from *B. garinii* (PBi). If this is correct, the sensitivity for the Liaison IgG assay may be lower in samples from Northern Europe where *B. afzelii* is the most prevalent infecting genospecies. However, a previous study [22] evaluating a recombinant *Borrelia* line immunoblot assay displayed the highest sensitivity for the recombinant VlsE of *B. garinii* (PBi) for both IgM and IgG detection. The study also showed that the most sensitive antigen for IgG in all LB stages, especially in early manifestations like EM and acute LNB, is VlsE followed by DbpA and p58 while VlsE of *B. afzelii* (PKo) reacted poorly with samples from patients with ACA and LA (late manifestations). The poor reactivity for LA might be explained by the rare observation of *B. burdorferi* sensu stricto (s.s.) in ticks and patients from Northern Europe [22]. However, cross reactivity of VlsE between different species may occur and different species are more likely to cause certain clinical signs and symptoms, e.g., *B. garinii* has been associated with more distinct symptoms and more pronounced intrathecal inflammation in LNB while *B. afzelii*, in Europe, is often associated with skin manifestations like EM and ACA [23, 24]. In Europe, there are at least five different species that are known to be pathogenic to humans [25]. A previous study has shown a higher specificity for the Enzygnost assay in both IgM and IgG compared to the Liaison assay [26], which is in line with our findings, especially for IgM indicating that recombinant VlsE antigens obtained from all three *B. burgdorferi* genospecies pathogenic to humans improved the diagnostic sensitivity with sustained specificity of LB. Most of the diagnostic assays in this study include VlsE as antigen. VlsE epitopes provoke an early antibody response, which is not detectable in ELISAs prepared from whole-cell sonicates of cultured *B. burgdorferi* bacteria, since the VlsE antigen is not expressed by the bacteria in vitro [27]. This present study shows that there is no gain in sensitivity, except for the Enzygnost IgM assay, analyzing the samples with both IgM and IgG if VlsE is used as antigen in the IgG test. The use of IgM may instead result in specificity problems. However, if excluding IgM testing is considered, the serodiagnostic IgG assay should include either shared antigens or antigens from the different pathogenic species.

The Recomwell IgG assay follows a principle of using a panel of several recombinant antigens in the same ELISA assay (p100, OspC, VlsE, p18), with a purpose of increasing the the sensitivity. However, in this study, there was a lower specificity without noticable gain in sensitivity for this assay and the RecomWell IgG assay has low screening value (AUC < 0.50) in consecutive patients with other diseases. The low specificity of the Recomwell IgG assay implicates that if used, it would be advisable to use a second confirmatory assay like another ELISA or an immunoblot.

When using blood donors as controls, awareness of seropositivity rate in the local population is crucial as this may be used as a pointer in clinical interpretation of the results, especially in patients with typical symptoms. However, it is of less importance if the specificity of the assay is high [13]. The seroprevalence in blood donors in this study is in agreement with previous studies done in Kalmar [11, 28], a region closely located to Jönköping County, indicating an increase in seroprevalence in the healthy population over the years. It is known that a high seroprevalence for both IgM and IgG can be found in a healthy population in *Borrelia* endemic areas which is in line with the results in this study.

This study included two control groups, blood donors and patients with other diseases. The results show that healthy blood donors consistently lead to higher specificity than controls with other diseases. The high seropositivity among patients with other diseases is caused by the fact that patients under investigation for symptoms that could be attributed to a tick-borne infection were referred to the specialized centers for further investigation (e.g., a lumbar puncture) partly due to their seropositivity and that the presence of antibodies in this case does not prove the occurrence of an active infection or disease, since antibodies, especially IgG, may persist for 10–20 years at least [29]. However, it cannot be excluded that some of the positive results reflect on-going LB, but in the referral center, seropositivity in serum is of little diagnostic value. In a systematic review by *Leeflang* et al. [6], it is recommended that “Future diagnostic accuracy studies should be prospectively planned cross-sectional studies, done in settings where the test will be used in practice”. This study follows these recommendations using patients referred for suspected LB, later classified as patients with other diseases. Thus, a future prospectively planned cross-sectional study should collect samples in the flow of patients at the time of the first suspicion of LB, not after referral of the patient. This is, however, hardly feasible to carry out for practical reasons, as recruitment should be done in primary care involving a large number of clinics.

We are aware that exclusion of the patient group with suspected LB (*n* = 24) in the statistical analyses may have resulted changed estimated test performances. But we would not know if they should be included in the patient group or the control group. Another drawback in the study population is that it did not include children. Examining diagnostic performance of the assays in paediatric patients would have been of interest, since the seroprevalence may differ from adults and children often present with neurological symptoms early in the course of LNB, when antibody production is low and hard to detect and laboratory diagnosis therefore remain uncertain [15, 30] .

## Conclusions

The IgG detection kits showed comparable results with small variations both within and between assays, an average sensitivity 88% (range 85–91%) compared to IgM which showed lower sensitivities of 59% (range 50–67%), while the intra- and inter-assay results for IgM were more heterogeneous. Our findings support that separate IgM testing in serum from adult patients gives no added diagnostic value in LB diagnostics, especially in highly endemic areas, when modern IgG assays containing VlsE antigens from several of the main pathogenic species are used. However, the study showed that the Enzygnost IgM assay had a higher sensitivity and specificity which is of interest particularly in diagnosis of young children. The more suitable control group in this study consisted of samples from blood donors, since the high seropositivity rate found in patients assessed not to have LB, indicated that many of the patients investigated for suspected LNB were referred for further investigation partly due to the seropositivity found in serum, thus resulting in a study selection bias.
